# The role of systemic inflammatory markers in differentiating tuberculosis from nontuberculous mycobacterial colonization and infection: A retrospective observational study

**DOI:** 10.1097/MD.0000000000044861

**Published:** 2025-10-03

**Authors:** Savaş Gegin, Ahmet Cemal Pazarli, Burcu Özdemir, Esra Arslan Aksu, Levent Özdemir

**Affiliations:** a Samsun Training and Research Hospital, Chest Diseases Clinic, Samsun, Turkey; b Department of Pulmonary Diseases, Tokat Gaziosmanpaşa University Faculty of Medicine, Tokat, Turkey; c Department of Chest Diseases, Samsun University Faculty of Medicine, Samsun, Turkey.

**Keywords:** nontuberculous mycobacteria, systemic inflammatory indices, tuberculosis

## Abstract

Culture confirmation for tuberculosis (TB) and nontuberculous mycobacteria (NTM) is time-consuming, potentially delaying diagnosis and treatment. Identifying systemic inflammatory markers from routine blood tests may provide supportive information for differentiating TB from NTM infection or colonization. This study evaluates the diagnostic value of systemic inflammatory indices in distinguishing TB from NTM infection and colonization. This retrospective study included 480 patients diagnosed between January 2018 and December 2023. TB and NTM diagnoses were confirmed according to microbiological and clinical criteria. Hematological parameters, including neutrophil-to-lymphocyte ratio, platelet-to-lymphocyte ratio, lymphocyte-to-monocyte ratio, lymphocyte-to-C-reactive protein ratio, systemic immune-inflammation index (SII), and systemic inflammation response index (SIRI), were analyzed. Receiver operating characteristic analysis was performed for parameters showing statistical significance in differentiating TB from NTM colonization. Among the inflammatory markers assessed, lymphocyte count and mean platelet volume were significantly higher in TB compared with NTM colonization, while SII and SIRI indices were significantly lower. Receiver operating characteristic analysis identified optimal cutoff values for lymphocyte count (1.895; sensitivity 57.7%, specificity 71.8%) and SII (2.345; sensitivity 73.1%, specificity 69.4%). The 95% confidence intervals for the area under the curve values are presented in table and figures. Lymphocyte count, mean platelet volume, SII, and SIRI show potential as supportive diagnostic markers for differentiating TB from NTM colonization. These indices may aid clinical decision-making while awaiting culture results; however, further studies with larger sample sizes and prospective validation are warranted.

## 1. Introduction

Tuberculosis (TB), caused by the *Mycobacterium tuberculosis* complex (MTC), remains a significant global public health issue. Although its incidence is gradually declining, TB continues to cause substantial morbidity and mortality, particularly among immunosuppressed individuals and those infected with Human Immunodeficiency Virus.^[1]^ TB is a treatable and preventable disease that primarily affects the lungs, although it may also involve other organs. In the diagnosis of pulmonary TB, clinical history, physical examination findings, and chest radiography typically raise suspicion, while bacteriological or histopathological methods are used for confirmation.^[2]^

Nontuberculous mycobacteria (NTM) are opportunistic microorganisms commonly found in environmental sources such as soil and water. In recent years, the incidence and clinical relevance of NTM infections have increased. While they may exist as colonizers in individuals without risk factors, NTMs are more likely to cause pulmonary diseases similar to TB, especially in elderly individuals, those with underlying structural lung disease, or patients with immunosuppression. The diagnosis requires clinical, radiological, and microbiological evidence.^[3]^

Differentiating MTC and NTM infections remains a diagnostic and therapeutic challenge. Both bacterial species show acid-fast staining resistance. Although culture examination is required for definitive diagnosis, medications used in the treatment of MTC are generally ineffective against NTM infections. In cases involving NTM, false-positive results may occur with acid-fast staining, making it necessary to confirm with culture. This diagnostic overlap may lead to inappropriate treatment, delayed therapy, and antimicrobial resistance when culture results are pending.^[4]^

Understanding the role of systemic inflammation in TB and NTM colonization or infection may provide insight into disease progression and prognosis. In recent years, various readily calculable peripheral blood markers of systemic inflammation and immune response have been investigated across numerous conditions, including malignancies, ulcerative colitis, Crohn disease, stroke, sepsis, chronic obstructive pulmonary disease (COPD), and pulmonary embolism. Ratios such as the neutrophil-to-lymphocyte ratio (NLR), platelet-to-lymphocyte ratio (PLR), systemic immune-inflammation index (SII), and systemic inflammation response index (SIRI) have shown potential for prognostic utility.^[5–10]^ Similarly, studies have suggested that NLR and PLR could be used to assess disease severity and treatment response in patients with TB.^[11,12]^

The present study aims to investigate systemic inflammatory markers in patients diagnosed with TB and those with NTM colonization or infection, and to explore whether these markers may aid in differentiating TB from NTM.

## 2. Materials and methods

**Figure 1.**
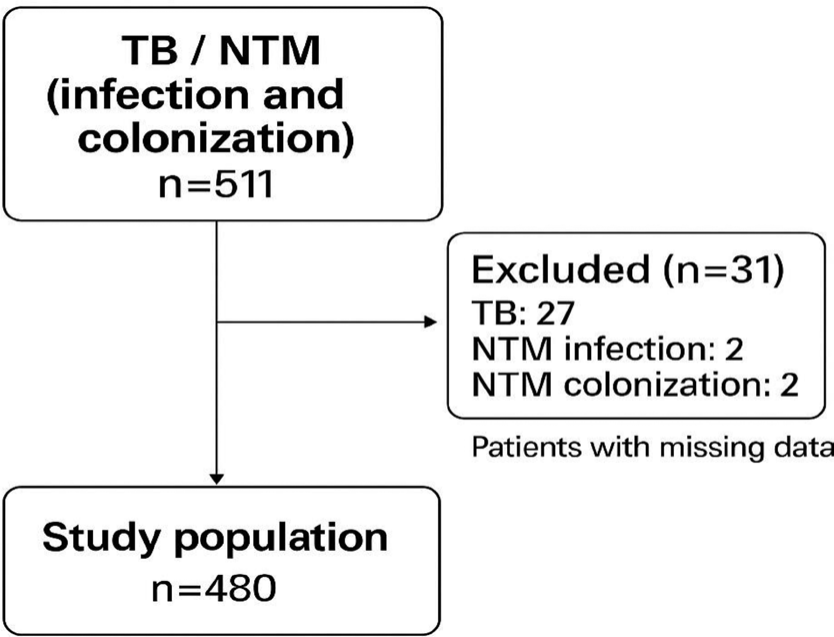
Study population.

This retrospective study was conducted in the Department of Pulmonology at Samsun Training and Research Hospital and included 511 patients with culture-confirmed MTC or NTM between January 2018 and December 2023. Data could not be retrieved for 31 patients (TB: n = 27; NTM infection: n = 2; NTM colonization: n = 2). Patients with missing data for any of the parameters analyzed were excluded from the relevant statistical analyses (listwise deletion), resulting in a final study population of 480 patients (Fig. 1).

*TB diagnosis*: patients with MTC growth detected in sputum, bronchoalveolar lavage, or tissue cultures (cultured using the BACTEC 460 TB system).^[13]^

*NTM colonization diagnosis*: NTM growth in a single sputum culture.^[14]^

*NTM infection diagnosis*: The presence of respiratory or systemic symptoms along with radiological findings (e.g., cavities, nodules, bronchiectasis) Additionally:

1. Growth in at least 2 sputum cultures collected at different times, or
2. Growth in a bronchoalveolar lavage culture, or
3. Histopathological evidence of mycobacterial infection (e.g., granulomatous inflammation or acid-fast bacilli [AFB]) from transbronchial or other lung biopsies, along with a positive NTM culture, or histopathological evidence plus at least one culture-positive sputum or bronchial washing for NTM.^[14]^

Demographic data and laboratory parameters were obtained from the hospital information system. All hemogram and C-reactive protein (CRP) analyses were performed by laboratory personnel who were blinded to the patients’ clinical diagnoses. At the time of diagnosis, the following parameters were calculated using the hemogram WBC, hemoglobin, hematocrit, platelets, neutrophils, lymphocytes, monocytes, mean platelet volume (MPV), platelet distribution width, red cell distribution width-coefficient of variation, CRP, and NLR, PLR, lymphocyte-to-monocyte ratio, lymphocyte/CRP ratio lymphocyte-to-C-reactive protein ratio, SIRI, and SII. All hematology and CRP measurements were performed using the same automated analyzers and standardized laboratory protocols throughout the study period. Inter-assay variability was regularly monitored and was within the acceptable range for clinical laboratory practice (coefficient of variation < 5%).

The study was approved by the Samsun University Non-Interventional Clinical Research Ethics Committee (Decision No: 2023/10/12, Date: 24/05/2023). The requirement for informed consent was waived by the committee due to the retrospective nature of the study and anonymization of patient data.

## 3. Results

Data from 480 patients (mean age 54 ± 17.4 years) diagnosed between January 2018 and December 2023 were analyzed. The majority were male (76.7%), and most had TB (87.1%), followed by NTM infection (7.5%) and NTM colonization (5.4%).

As shown in Table 1, TB patients exhibited a stronger systemic inflammatory response compared with those with NTM colonization, reflected by lower lymphocyte counts and higher MPV, SII, and SIRI values (*P* < .05 for all). In contrast, other inflammatory markers did not show statistically significant differences between TB and NTM groups, suggesting a substantial overlap in their systemic inflammation profiles when active infection is present.

**Table 1:** Association of biomarkers with TB and NTM.

	TB n = 480	NTM colonization n = 26	NTM infection n = 36	*P*
WBC (×10^9^/L)	8.98 ± 3.07	8.28 ± 2.49	10.72 ± 7.75	.169
Hemoglobin (g/dL)	12 ± 2.1	12.1 ± 2.2	11.9 ± 2.6	.933
Hematocrit (%)	36.4 ± 5.6	37.4 ± 4.6	37.1 ± 5.4	.546
Platelets (×10^9^/L)	333.9 ± 146.7	292.9 ± 145.1	311.9 ± 112.7	.275
Neutrophils (×10^9^/L)	6.49 ± 2.75	5.40 ± 2.27	8.14 ± 7.66	.036
Lymphocyte (×10^9^/L)	**1.55 ± 0.78**	**2 ± 0.81**	**1.66 ± 0.60**	.**012**
Monocyte (×10^9^/L)	0.69 ± 0.43	0.61 ± 0.23	0.72 ± 0.37	.581
MPV (fL)	**7.83 ± 1.58**	**8.67 ± 1.39**	**8.41 ± 1.48**	.**005**
PDW (%)	15.40 ± 3.31	15.20 ± 4.05	14.34 ± 3.54	.193
RDVCV (%)	16.08 ± 7.30	15.05 ± 2	15.57 ± 3.37	.712
CRP (mg/L)	64.77 ± 63.08	42.71 ± 47.13	68.85 ± 80.29	.245
Sedimentation (mm/h)	59.52 ± 30.96	40.35 ± 24.33	52.56 ± 30.44	.025
NLR (unitless)	5.70 ± 5.28	3.58 ± 3.31	6.13 ± 8.02	.134
LMR (unitless)	3.01 ± 3.94	3.62 ± 1.80	2.62 ± 1.27	.582
PLR (unitless)	298.11 ± 385.05	164.31 ± 98.51	220.50 ± 136.50	.103
LCRPR (unitless)	0.20 ± 0.70	0.23 ± 0.31	0.16 ± 0.33	.923
SIRI (unitless)	**3.66 ± 3.31**	**1.99 ± 1.37**	**4.85 ± 7.51**	**.000**
SII (unitless)	**1896.29 ± 1943.41**	**996.75 ± 980.08**	**2195.36 ± 3935.22**	**.000**

Bold values indicate statistically significant values.

CRP = C-reactive protein, LCRPR = lymphocyte-to- C-reactive protein ratio, LMR = lymphocyte-to- monocyte ratio, MPV = mean platelet volume, NLR = neutrophil-to-lymphocyte ratio, NTM = nontuberculous mycobacteria, PDW = platelet distribution width, PLR = platelets-to-lymphocyte ratio, RDVCV = red cell distribution width, SII = systemic immune-inflammation index, SIRI = systemic inflammatory response index, WBC = white blood count.

**Figure 2.**
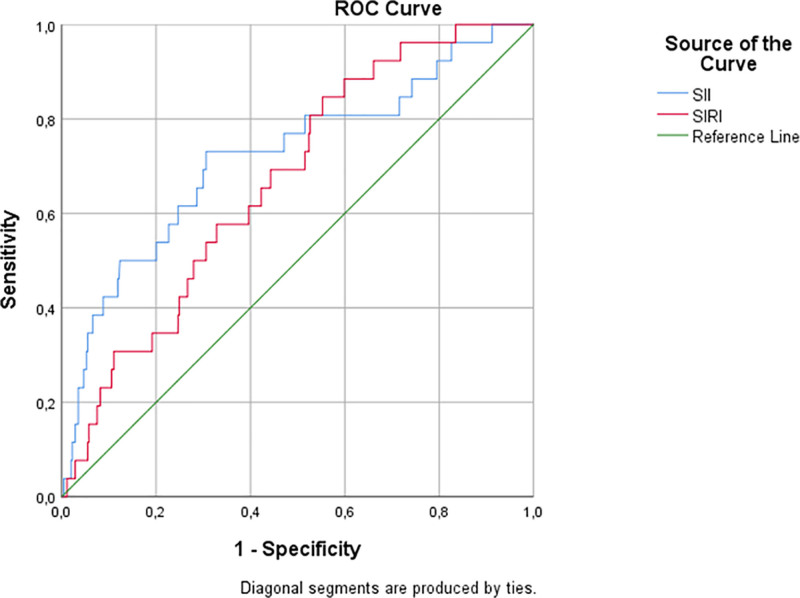
Receiver operating characteristic (ROC) curves of SII and SIRI in predicting TB and NTM colonization, with the 95% confidence intervals (CI) for the AUC values provided in Table 2. AUC = area under the curve, NTM = nontuberculous mycobacteria, SIRI = systemic inflammation response index, TB = tuberculosis.

**Figure 3.**
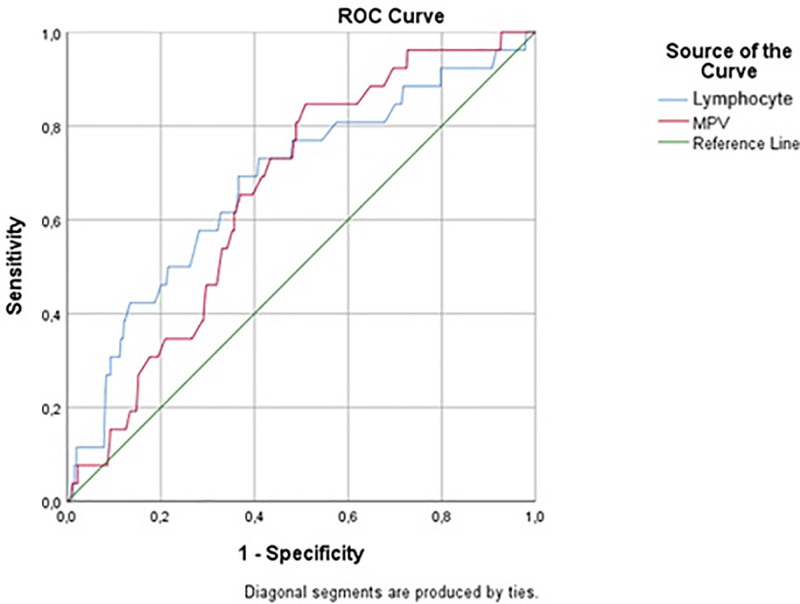
Receiver operating characteristic (ROC) curves of lymphocyte count and MPV in predicting TB and NTM colonization, with the 95% confidence intervals (CI) for the AUC values provided in Table 2. AUC = area under the curve, MPV = mean platelet volume, NTM = nontuberculous mycobacteria, TB = tuberculosis.

Diagnostic performance analysis (Table 2 and Figs. 2 and 3) indicated that lymphocyte count and SII were the most promising parameters for differentiating TB from NTM colonization, both demonstrating moderate sensitivity and specificity. MPV and SIRI also showed significant but slightly lower discriminative value. These trends emphasize the potential of these indices as supportive (not standalone) tools in distinguishing colonization from active disease.

**Table 2:** Diagnostic accuracy of biomarkers in predicting TB and NTM colonization.

	AUC	95% CI	Cutoff	*P*	Sensitivity (%)	Specificity (%)
Lymphocyte (×10^9^/L)	0.678	0.566–0.790	1895	.002	57.7	71.8
MPV (fL)	0.659	0.568–0.750	8160	.006	65.4	63
SIRI (unitless)	0.670	0.577–0.763	2345	.004	69.2	55.7
SII (unitless)	0.722	0.609–0.834	887.66	.000	73.1	69.4

AUC = area under the curve, MPV = mean platelet volume, NTM = nontuberculous mycobacteria, SII = systemic immune-inflammation index, SIRI = systemic inflammatory response index.

## 4. Statistical analysis

Variables included in the receiver operating characteristic (ROC) analysis were selected if they demonstrated statistical significance in univariate analysis (*P* < .05) and/or had established clinical relevance in differentiating TB from NTM, as supported by previous literature.

All data were analyzed using SPSS version 23 for Windows (SPSS Inc., Chicago). Frequencies and percentages were calculated for categorical variables, and means with standard deviations for continuous variables. Normality and homogeneity of variance were assessed using the Kolmogorov–Smirnov and Levene tests, respectively. For normally distributed variables, one-way ANOVA and Bonferroni post hoc tests were used; for non-normally distributed variables, Welch ANOVA and Tamhane post hoc tests were applied. The predictive power of lymphocyte count, MPV, SII, and SIRI in distinguishing TB from NTM was evaluated using the area under the curve of the ROC analysis.

## 5. Discussion

This retrospective study examined the diagnostic differential value of systemic inflammatory markers in patients with TB, NTM infection, and NTM colonization. The findings demonstrate that certain parameters, particularly lymphocyte levels, MPV, SII, and SIRI, show statistically significant differences in distinguishing TB from NTM colonization.

In our literature review, we identified various studies where systemic inflammatory markers were used in the differential diagnosis of TB and nontuberculous lung diseases such as pneumonia and sarcoidosis, as well as in evaluating the response to TB treatment.^[10,12,15–19]^ To the best of our knowledge, our study is the first to evaluate these parameters in the differentiation between TB and NTM colonization and infection.

Berhane et al evaluated data from 146 TB and community-acquired bacterial pneumonia patients and found that NLR and sedimentation rate were significantly higher in TB patients.^[12]^ Conversely, in a study by Yoon et al involving 122 TB and 94 community-acquired bacterial pneumonia patients, NLR was found to be significantly lower in TB patients.^[10]^ Similarly, in a meta-analysis of 10 studies by Shojaan et al, involving 730 TB and 725 community-acquired pneumonia patients, NLR levels in TB patients were reported to be lower compared to those with pneumonia, suggesting its potential utility in differential diagnosis.^[15]^ In a study by Yu et al, which evaluated 1327 TB patients and 703 patients with nontuberculous pulmonary infections, SII index and fibrinogen levels were found to be significantly higher in TB patients. Moreover, these parameters were also higher in smear-positive TB patients compared to smear-negative ones.^[16]^ In the study by Jeon et al., which evaluated 220 healthy individuals, 110 TB patients, and 159 patients with nontuberculous pulmonary infections, the SIRI index was shown to be more useful than NLR in differentiating TB from non-TB pulmonary infections.^[17]^ In a study by Chai et al involving 60 smear-negative TB and 70 patients with nontuberculous pulmonary infections, NLR (73.3% sensitivity, 75.7% specificity; cutoff: 2.74), PII (78.3% sensitivity, 82.8% specificity; cutoff: 0.20), and SIRI (80% sensitivity, 77.1% specificity; cutoff: 0.97) were found to be significantly higher.^[18]^

In a study by Iliaz et al, involving 51 TB patients, 40 patients with sarcoidosis, and 43 healthy individuals, the ideal cutoff value for NLR in differentiating TB from sarcoidosis was determined to be 2.55, with 79% sensitivity and 69% specificity.^[19]^

Systemic inflammatory markers have also been used in latent TB infection, in evaluating treatment response, and in the differentiation of cavitary lesions.^[20–22]^ In a study by Huang et al involving 4938 individuals, NLR levels were found to be significantly lower in patients with latent TB infection (2.0 ± 1.0).^[20]^ In a study by Ştefanescu et al on 90 patients receiving treatment for pulmonary TB, assessments made in the second month of therapy showed that acid-resistant bacilli had turned negative in 63 patients and remained positive in 27. In acid-resistant bacilli-negative patients, significant reductions were observed in NLR, PLR, MLR, SII, and sedimentation rates, suggesting these parameters may be useful in monitoring treatment response.^[21]^ In a study by He et al involving 1233 patients, CRP, NLR, MLR, PLR, SII, and SIRI levels were found to be significantly higher in patients with cavitary pulmonary TB.^[22]^

In our study, lymphocyte levels were found to be lower and MPV levels higher in TB patients compared to those with NTM colonization. Additionally, the significantly higher SII and SIRI indices in TB patients support the presence of a more pronounced systemic inflammatory response during active infection. These findings are consistent with results reported in previous studies. Notably, parameters such as NLR, PLR, and SII have been shown in earlier research to be useful in assessing infection severity and treatment response in TB patients.^[21,22]^

The higher lymphocyte count in patients with NTM colonization suggests that this condition does not induce an active infection in the body and elicits a more limited immune response. According to the ROC analysis, a lymphocyte level cutoff value of 1.895 yielded a sensitivity of 57.7% and a specificity of 71.8%, indicating that this parameter has a certain diagnostic discriminative power. Similarly, the SII index, with a cutoff value of 2.345, demonstrated a sensitivity of 73.1% and specificity of 69.4%, suggesting that it may be a practical marker in differentiating TB from NTM colonization.

However, the other parameters evaluated in the study did not demonstrate significant discriminative power in differentiating NTM infection from TB. This suggests that both conditions can trigger systemic inflammation and that inflammatory markers alone may be insufficient for distinction. This finding supports the frequently emphasized necessity in the literature for a comprehensive clinical, radiological, and microbiological evaluation.

Our study has several limitations. Primarily, it was single-centered and retrospective in design. The lower number of patients with NTM colonization and infection compared to the TB group may have resulted in the absence of statistically significant differences for certain parameters. Moreover, since patients’ comorbidities were not excluded, the inflammatory parameters may have been confounded. In addition, systemic inflammatory marker levels can be influenced by concurrent infections, recent antibiotic use, and other inflammatory conditions, which were not excluded in our study and should be considered as potential confounders when interpreting the results. No internal validation (e.g., bootstrapping or cross-validation) was performed in this study. Furthermore, additional subgroup analyses within the NTM cohort were not conducted due to the limited sample size, which could have reduced statistical power. The absence of internal validation may affect the stability of the results and should be considered a limitation.

In conclusion, this study has shown that some systemic inflammatory indices calculated from peripheral blood parameters may be helpful, particularly in distinguishing TB from NTM colonization. However, it should be kept in mind that these markers alone are not diagnostic and should be interpreted as supportive clinical data. Prospective studies with larger sample sizes will more clearly demonstrate the diagnostic reliability of these markers.

## Author contributions

**Conceptualization:** Savaş Gegin, Burcu Özdemir, Levent Özdemir.

**Data curation:** Savaş Gegin, Burcu Özdemir, Esra Arslan Aksu, Levent Özdemir.

**Formal analysis:** Savaş Gegin, Burcu Özdemir, Esra Arslan Aksu, Levent Özdemir.

**Funding acquisition:** Savaş Gegin, Ahmet Cemal Pazarli, Burcu Özdemir, Levent Özdemir.

**Investigation:** Savaş Gegin, Ahmet Cemal Pazarli, Burcu Özdemir, Levent Özdemir.

**Methodology:** Savaş Gegin, Ahmet Cemal Pazarli, Burcu Özdemir, Esra Arslan Aksu, Levent Özdemir.

**Project administration:** Savaş Gegin, Ahmet Cemal Pazarli, Burcu Özdemir, Esra Arslan Aksu, Levent Özdemir.

**Resources:** Savaş Gegin, Burcu Özdemir, Levent Özdemir.

**Software:** Savaş Gegin, Ahmet Cemal Pazarli, Burcu Özdemir, Esra Arslan Aksu, Levent Özdemir.

**Supervision:** Savaş Gegin, Burcu Özdemir, Esra Arslan Aksu, Levent Özdemir.

**Validation:** Savaş Gegin, Ahmet Cemal Pazarli, Burcu Özdemir, Levent Özdemir.

**Visualization:** Savaş Gegin, Ahmet Cemal Pazarli, Burcu Özdemir, Levent Özdemir.

**Writing – original draft:** Savaş Gegin, Burcu Özdemir, Esra Arslan Aksu, Levent Özdemir.

**Writing – review & editing:** Savaş Gegin, Ahmet Cemal Pazarli, Burcu Özdemir, Esra Arslan Aksu, Levent Özdemir.
